# Problematic Internet Use and Perceived Quality of Life: Findings from a Cross-Sectional Study Investigating Work-Time and Leisure-Time Internet Use

**DOI:** 10.3390/ijerph17114056

**Published:** 2020-06-06

**Authors:** Lingling Gao, Yiqun Gan, Amanda Whittal, Sonia Lippke

**Affiliations:** 1 Department of Psychology & Methods, Jacobs University Bremen, Campus Ring 1, 28759 Bremen, Germany; l.gao@jacobs-university.de (L.G.); a.whittal@jacobs-university.de (A.W.); s.lippke@jacobs-university.de (S.L.); 2 School of Psychological Cognitive Sciences, and Beijing Key Laboratory of Behavior and Mental Health, Peking University, Beijing 100871, China

**Keywords:** internet addiction, work, leisure, stress, quality of life, smoking

## Abstract

Avoiding the potential negative impact brought by problematic internet use is becoming more important. To better understand public health and addiction, this study investigated to what extent work-time and leisure-time internet use relate to problematic internet use and perceived quality of life among college students and highly educated adults. An online cross-sectional survey with 446 individuals was assessed in Germany. Linear regression analyses were used to predict problematic internet use. Ordinal regression analyses were applied to predict perceived quality of life. Results showed that leisure-time internet use, but not work-time internet use, was positively associated with problematic internet use. Participants whose work-time internet use could be considered balanced (5–28 h/week in this study) indicated a higher perceived quality of life compared to individuals with little or large amount of internet use for work. The findings still emerged when taking negative feelings, perceived stress, smoking status and alcohol consumption into account. As both work-time and leisure-time internet use can be risk factors for mental health in terms of problematic internet use and perceived quality of life, well-controlled internet use rather than excessive use is recommended. This should be kept in mind when dealing with the Coronavirus pandemic and its aftermath.

## 1. Introduction

Understanding health and well-being in the face of digitalization requires an investigation of internet use and whether it can be problematic in terms of leading to addiction. In modern times, with a substantial amount of people using the internet for work, studies, and entertainment, the internet fundamentally shapes people’s daily experiences, including perceived quality of life [1,2,3]. 

Having a good quality of life is important for numerous reasons, and is a key determinant of public health. The judgment of ’perceived quality of life’, which is suggested to represent how satisfied people are with their present state of affairs, is based on a comparison with a standard that people set for themselves [4]. Many factors can contribute to improving or worsening quality of life. It has been found that psychological domains such as negative feelings [5] and perceived stress [6] are negative indicators of quality of life. Moreover, health-risk behaviors such as smoking are negatively related to perceived quality of life [7]. Alcohol consumption status has shown mixed results. Some findings have shown that addiction to alcohol is negatively related to quality of life [8], while it failed to reveal correlates with perceived quality of life in other studies [9,10]. Furthermore, it has been found that regular alcohol consumption is associated with increased quality of life [11,12]. 

Research has also shown that the internet can help people to obtain a higher perceived quality of life by promoting their work, education, and communication [13]. Some studies have pointed out that apps are acceptable and easy to use, providing health communication and education to populations with low health literacy [14]. It also has been found that the internet can improve perceived quality of life by providing mental stimulation and assistance [15]. Researchers have also, however, reported that people who spend large amounts of time on the internet may suffer as a result [16,17], which can decrease perceived quality of life [18]. Consequences of this include suffering from long-term lack of sleep, deterioration of physical health, difficulties concentrating on work, and a reduction in close relationships with family members [19].

Problematic internet use can be considered a risk factor for reduced quality of life, and has therefore been investigated during the past twenty years [1,20,21]. Adolescents and young adults are at particularly high risk of problematic internet use [17,22]. Males are more likely to have problematic internet use than females in some studies [23], however, other studies have reported no significant gender difference [24]. Besides findings on demographic variables, previous studies have also found that problematic internet use is associated with potential addictive habits such as smoking, alcohol or coffee consumption, and taking drugs [25]. However, while some researchers have argued that alcohol and other illegal drugs are only linked to problematic internet use in tobacco users [26], others have found that smoking status is not related to problematic internet use [27].

Despite the negative consequences of problematic internet use, the internet is of necessity for many people in modern times, especially for particular groups. For example, college students and highly educated adults use the internet more frequently than other populations [28], because they require it for work/study purposes, as well as for leisure time in their daily life. To better make use of the advantages and avoid the potential risks brought by internet use, many studies have investigated specific online activities, such as gaming, social networking, and online gambling, which have been found to lead to problematic internet use [29]. Moreover, it has been found that only considering the amount of time spent online as a judgment of problematic internet use is questionable and insufficient to make accurate judgments [30]. How work and leisure internet use may each contribute to problematic internet use remains unclear, as well as the potential consequences on perceived quality of life. Thus, the purpose of this study is to explore the independent contributions of work and leisure internet use to problematic internet use, and the corresponding impact on perceived quality of life. The variables and theoretical framework used to explore this are outlined below.

Internet use to serve work and study purposes, is called “work-time internet use” in this study. All internet use that indicates leisure purposes (e.g., shopping online, playing games), regardless of whether it occurs during or after working hours, is regarded as “leisure-time internet use” in this study. Given the resources and opportunities offered by the internet, organizations have encouraged people to use the internet to work more productively and efficiently [31], which provides the possibility for work-time internet use. However, studies have found that employees also have increased opportunities to use the internet for leisure purposes during or after work hours, which may also be overwhelming [32]. In other words, a higher quantity of internet use for the two different qualities harbors the risk of an excessive amount, and with that becoming unbalanced with other behaviors.

This interrelation of different behaviors such as work-time and leisure-time internet use can be understood on the basis of the compensatory carry-over action model (CCAM) [33]. According to the CCAM, different behaviors are not isolated; rather, they interact with each other in complex ways. The model posits that emotionally relevant higher-level goals initiate intentions to engage in behaviors. To achieve a higher-lever goal, in this case, for example, good perceived quality of life, individuals may integrate the internet in their work and life (e.g., for social connection). Cognitive factors such as self-efficacy influence this process, as they make a difference in how people think and are able to motivate themselves. Both internet use for work and leisure are associated with internet use self-efficacy, which has been found to be a potentially important factor in explaining individuals’ decisions on internet use [34,35]. Stress management also influences this process thorough cognitive factors. Problematic internet use has been found to be related to perceived stress and negative feelings, such as depression and anxiety [36]. According to the model, the process of work-time and leisure-time internet use interrelate via compensatory cognitions and carry-over mechanisms. A person might carry over resources from one type of internet use to another, including experiences, skills, and cognitions. The outcomes such as perceived quality of life and problematic internet use may relate to both work-time internet use and leisure-time internet use. 

The main aim of this study is to understand the potential mechanisms through which problematic internet use might be decreased and quality of life be improved, by investigating the different contributions of work-time and leisure-time internet use. In this process, as the CCAM indicates, other factors such as negative feelings, perceived stress, smoking status, and alcohol consumption are also investigated. Accordingly, this study examines the following research questions among college students and highly educated adults:

(1) Do work-time and leisure-time internet use interrelate with problematic internet use?
(2) Do work-time and leisure-time internet use interrelate with perceived quality of life?
(3) Are there differences in the relationships when additionally taking negative feelings, perceived stress, smoking status, and alcohol consumption into account?

## 2. Materials and Methods

### 2.1. Participants

A total of 513 participants completed an online survey. After excluding invalid data (e.g., the sum of work-time internet use and leisure-time internet use was larger than 168 h/week (h/w)), the current study included 446 participants (59.6% female, *n* = 266) whose ages ranged from 17 to 77 (M = 25.8, SD = 11.6). Among the sample, 232 (52%) participants achieved a bachelor level and above education, 214 (48%) participants graduated from high school (or had an equivalent education level) and were currently studying at a university. Most of the study participants (97.3%, *n* = 434) reported being employed or studying. According to the problematic internet use test, 29.4% participants (*n* = 131) who scored high (problematic internet use ≥ 50) had problematic internet use, and the other 70.6% participants (*n* = 315) who scored low (problematic internet use < 50) had normal internet use levels. Regarding perceived quality of life, 10.5% of participants (*n* = 47) reported a very poor or poor level and 58.1% of participants (*n* = 259) reported a good or very good level.

### 2.2. Procedure

This cross-sectional study used an online questionnaire to collect data from October 2016 to July 2017 in Germany. The link for the survey was sent but not limited to the staff and students in universities. Participants were free to answer the questions by clicking the appropriate box (multiple choice questions) or input content (open questions). On the first page of the online survey, all participants were informed of the confidentiality and anonymity of their responses, and the people who clicked the box to provide their informed consent would continue to the questionnaire pages. The study was part of a project investigating the interrelationship between internet use and health behaviors, and received ethical approval by the Ethics Commission of the German Association of Psychology (Deutsche Gesellschaft für Psychologie, EK-A-SL022013).

### 2.3. Measurements

Work-time and leisure-time internet use were assessed separately, by asking participants questions about their internet use in a typical week. One question (a) “How many days did you spend on this (work/study, leisure) per week?”, was followed by question (b) “How many hours on average did you spend on the days you did it?”. The time spent on internet use for work and leisure, separately, were obtained from the multiplied scores of questions (a) and (b). This instrument has been shown to have acceptable reliability and validity for measuring the time spent on internet use [37,38,39].

In order to identify the differences of problematic internet use and perceived quality of life between different work-time/leisure-time internet users, participants were put into categorical quartiles work-time/leisure-time internet use groups: work-time internet use groups 1, 2, 3, 4 (WG1, WG2, WG3, WG4); leisure-time internet use groups 1, 2, 3, 4 (LG1, LG2, LG3, LG4) (see Table 1 for details). These categories are not derived from, but are also in line with previous studies that have shown work-time ≥ 55 h/w to be harmful with health [40]. Leisure-time internet use should be no more than 10 h/w, and ≥30 h/w may lead to health-risk behaviors such as sleep problems [41].

Problematic internet use was measured on a 4-point Likert scale with 10 items [42]. The example question was “My thoughts are constantly around the internet, even when I’m not online”, and was scored 1–4 from “completely disagree” to “agree completely”. Suggested by the scale, the total score range of this scale was 20–80, and it was distinguished by three types of internet use: no internet addiction (20–49), internet addiction tendency (50–59), internet addiction (60–80). In this study, the participants whose scores ≥ 50 were considered to have problematic internet use [42]. Cronbach’s alpha in this study was 0.84.

Perceived quality of life was measured by asking “Please think about the last four weeks: How would you rate your perceived quality of life?” with a 5-point scale ranging from “very poor” to “very good” [43]. The scores of the participants were tripartite categorized as very poor/poor, neither poor nor good, good/very good. This single-item measure assessing self-rated perceived quality of life has been found to have acceptable reliability and validity in previous surveys [44,45,46].

Negative feelings were measured by asking “In the last months, how often do you have negative feelings such as blue mood, despair, anxiety, depression in the last four weeks?” with a 5-point scale ranging from “never” to “always” [43]. This single-item measure assessing negative feelings has been found to have acceptable reliability and validity in previous surveys [47,48].

Perceived stress was measured with two questions from the perceived stress scale [49]: “How often do you feel nervous and stress?”, and “How often do you feel difficulties were piling up so high that you could not overcome them?”, and rated on a 5-point Likert scale. The split-half reliability of this scale was satisfactory (0.73 according to the Spearman Brown formula, the equivalent of Cronbach’s alpha for two-item scales [50]).

Smoking status was measured by asking the participants “Are you a smoker?”, and participants who answered “nonsmoker” or “ex-smoker” scored 1 point, while participants who answered “occasional smoker” or “regular smoker” scored 0. This question has been found to have acceptable reliability and validity in previous surveys [51,52]. 

Alcohol consumption status was measured by asking participants “How often do you drink alcoholic beverages?”, and participants who answered “nonregular drinker” or “seldom drinker (who drinks less than 1 times/week)” scored 1 point, while participants who answered “regular drinker (who drinks more than 1 time/week)” scored 0. These parameters were set up in accordance with a previous studies that defines a regular alcohol drinker as someone who drinks at least weekly [12]. This question has been found to have acceptable reliability and validity [53].

Participants also answered socio-demographic questions, such as age, gender, height, weight, marital status, employment status, and education level. Body mass index (BMI; kg/m^2^) was calculated using self-reported height and weight.

### 2.4. Statistical Analysis

Bivariate correlation analyses were performed to examine the relationships between work-time and leisure-time internet use, problematic internet use and perceived quality of life. Linear regression analyses were used to predict problematic internet use by socio-demographic variables, work-time internet use, leisure-time internet use, negative feelings, perceived stress, smoking status, and alcohol assumption status. Ordinal regression analyses were used to predict perceived quality of life by socio-demographic variables, work-time internet use, leisure-time internet use, negative feelings, perceived stress, smoking status, and alcohol assumption status. Analysis of variance (ANOVA) was used to compare the mean differences of researched variables such as problematic internet use and perceived quality of life in different internet use groups. Analyses were performed with SPSS 26 (IBM Corp, New York, US). Significance was accepted at a *p-*level of 0.05.

## 3. Results

### 3.1. Descriptive Statistics

Descriptive statistics are presented in Table 1. The average internet use time during work was about 17.2 h/w. Participants also reported spending an average of 14.7 h/w online for leisure purposes.

### 3.2. Correlation Analyses in Main Study Variables

Bivariate Spearman correlation analyses were performed to examine the relationships between main research variables. The results showed a positive relationship between work-time internet use and leisure-time internet use, and a negative relationship between problematic internet use and perceived quality of life (Table 2).

### 3.3. Problematic Internet Use in Different Work-Time and Leisure-Time Internet Use Groups

To examine whether work-time and leisure-time internet use were interrelated with problematic internet use, when controlling for socio-demographic variables, linear regression analyses were performed with dummy coding for work-time internet use and leisure-time internet use. The findings are presented in Table 3, in which unstandardized B coefficients are reported but not beta. This is for reporting the value of problematic internet use of leisure-time (work-time) internet use groups 2, 3, 4 compared to that of group 1.

Work-time internet use was not significantly associated with problematic internet use after controlling for socio-demographic variables (model 1). LG2, LG3, and LG4 scored higher on the problematic internet use scale compared to LG1, respectively (model 1). When negative feelings and perceived stress were added into model 2, the results of work-time and leisure-time internet use predicting problematic internet use were similar to model 1. Moreover, perceived stress was positively related to problematic internet use (model 2). When smoking status and alcohol consumption were added in model 3, the results of work-time and leisure-time internet use predicting problematic internet use were still similar to model 1 and model 2. Work-time internet use was not significantly correlated with problematic internet use, while LG2, LG3, and LG4 scored higher on the problematic internet use scale compared LG1, respectively. Negative feelings and perceived stress showed positive relationships with problematic internet use, while smoking status and alcohol consumption were not significantly correlated with problematic internet use (model 3).

**Figure 1:**
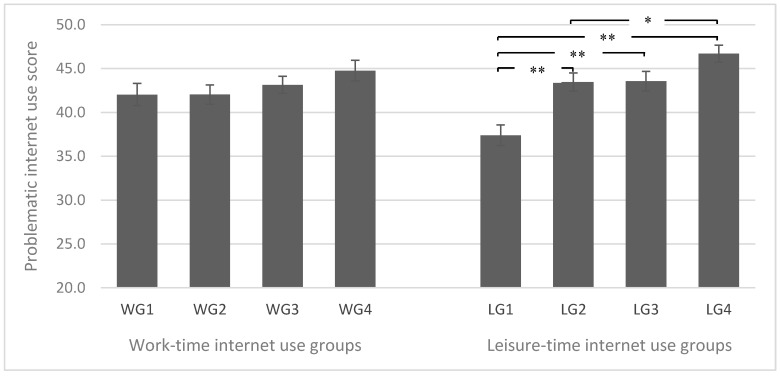
Means of problematic internet use in the four work-time internet use groups and four leisure-time internet use groups. WG1: work-time internet use group 1 (<5 h/w); WG2: work-time internet use group 2 (5–13.99 h/w); WG3: work-time internet use group 3 (14–27.99 h/w); WG4: work-time internet use group 4 (28–60 h/w); LG1: leisure-time internet use group 1 (<4 h/w); LG2: leisure-time internet use group 2 (4–10.99 h/w); LG3: leisure-time internet use group 3 (11–20.99 h/w); LG4: leisure-time internet use group 4 (21–84 h/w). * *p* < 0.05, ** *p* < 0.001.

To further investigate the differences of problematic internet use between leisure-time internet use groups, an ANOVA was performed. The results showed significant differences of problematic internet use between the four groups (F (1) = 33.28, *p* < 0.001). The paired comparisons of the mean differences of problematic internet use in the four leisure-time groups are shown in the right-hand side of Figure 1. LG1 with <4 h/w reported significantly lower score than LG2 with 4–10.99 h/w; LG2 with 4–10.99 h/w was not significantly different from LG3 with 11–20.99 h/w, but significantly lower than LG4 with 21–84 h/w in regard to problematic internet use. However, no such differences revealed to be significant for work-time internet use (Figure 1, left-hand side).

### 3.4. Perceived Quality of Life in Different Work-Time and Leisure-Time Internet Use Groups

To examine whether work-time and leisure-time internet use interrelated with perceived quality of life, when controlling for socio-demographic variables, ordinal regression analyses were performed with dummy coding for work-time internet use and leisure-time internet use. The findings are presented in Table 4.

The odds ratio (OR) value of reporting a good perceived quality of life in WG2 was 2.51 times compared with WG1 (χ^2^ (1) = 9.65, *p* < 0.05), after controlling for socio-demographic variables (model 1). The OR value of having a good perceived quality of life in WG3 was 1.85 times compared with WG1 (χ^2^ (1) = 5.13, *p* < 0.05), after controlling for socio-demographic variables (model 1). There was, however, no significant difference in perceived quality of life between WG4 and WG1. 

When controlling for negative feelings and perceived stress in model 2, the OR value of reporting a good perceived quality of life showed a similar pattern (χ^2^ (1) = 12.04, *p* < 0.001). Negative feelings and perceived stress showed significant negative relationships with perceived quality of life, respectively (model 2). 

**Figure 2:**
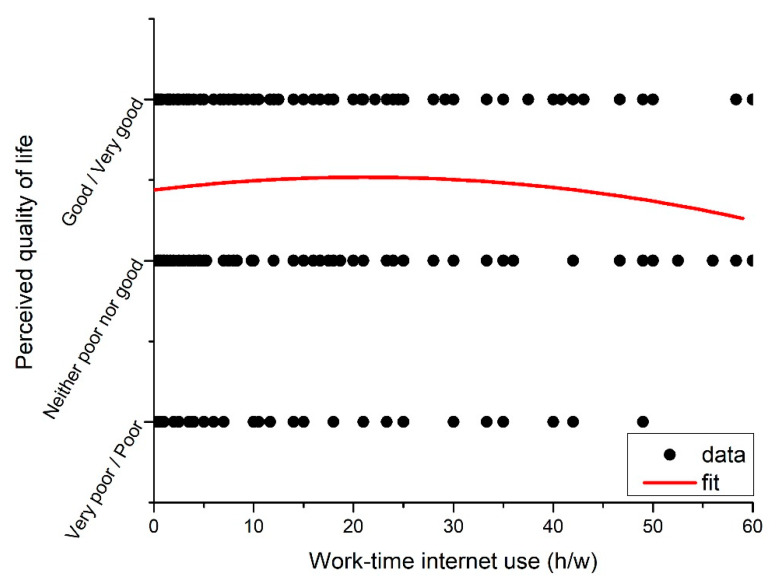
The scatterplot of perceived quality of life versus work-time internet use.

When the effect of smoking status and alcohol consumption were included in model 3, the OR value of reporting a good perceived quality of life in WG2 was 2.78 times compared with WG1 (χ^2^ (1) = 10.68, *p* < 0.001), the effect of WG3 in comparison to WG1 was close to significant. However, the second order fitting of the dependence of the perceived quality of life on the work-time internet use demonstrates that the high quality of life range is still located in the union of WG2 and WG3 (Figure 2). Taking this into consideration, participants in WG2 and WG3 were assumed have higher perceived quality of life compared to WG1. Moreover, negative feelings showed negative relationships with perceived quality of life (model 3). Regarding alcohol consumption, nonregular drinkers reported a lower OR value of good perceived quality of life compared with regular drinkers (model 3). Leisure-time internet use was not significantly correlated with perceived quality of life in any of the three models.

## 4. Discussion

Internet use is both essential for many individuals living and working in modern times, and poses potential risks of becoming a problematic behavior [1,20,21]. While studies have shown that long lengths of time spent using the internet can negatively impact quality of life [16,17], it has also been highlighted that the sole consideration of time spent online is questionable in determining problematic internet use [31]. While specific internet activities have been associated with problematic internet use [30], to our knowledge, types of internet use (i.e., whether used for work or leisure purposes) and their respective associations with problematic internet use and perceived quality of life have not been investigated systematically. As far as we know, this is the first study exploring problematic internet use and perceived quality of life when comparing the differences between work and leisure internet use separately. This comparison was assessed together with other factors (negative feelings, perceived stress, smoking, and alcohol assumption) that have been found to be imperative in understanding public health and addiction, among highly educated adults and college students. 

The results showed that problematic internet use was negatively correlated with perceived quality of life, which is in line with previous studies [1,54,55]. Work-time internet use and leisure-time internet use were correlated, however, they had different contributions to problematic internet use and quality of life. Leisure-time internet use but not work-time internet use was positively associated with problematic internet use. Participants whose work-time internet use was at a balanced level (between 5 to 28 h/w in this study) indicated a higher perceived quality of life compared to individuals with little or large amount of internet use for work. These results are largely consistent when taking negative feelings, perceived stress, smoking status, and alcohol assumption status into account. Negative feelings and perceived stress showed positive relationships with problematic internet use, respectively. Negative feelings showed a negative relationship with quality of life. Drinking alcohol on a regular basis showed a higher quality of life compared to nonregular drinkers.

### 4.1. Problematic Internet Use in Different Work-Time and Leisure-Time Internet Use Groups

One of the main findings of this study was that leisure-time internet use, but not work-time internet use, was strongly related to problematic internet use among college students and highly educated participants. After adjusting for socio-demographic variables, participants who use the internet for leisure purposes for 4–21 h/w scored around 4 points higher on problematic internet use, leaning more towards addictive behavior compared to participants who spent less than 4 h/w for leisure-time internet use. While we cannot make clinical conclusions about addiction per se, the higher problematic internet use may be a rough indication for potential addictive internet behavior. This finding is in line with and adds to previous studies, which reported that time spent online for recreational purposes is regularly observed in problematic internet use assessments [23,56,57,58]. 

Even though many college students and highly educated individuals have to use the internet for work purposes [59], and work-time internet use was higher than leisure-time internet use on average in this study, work-time internet use did not show a significant association with problematic internet use after controlling for socio-demographic variables. This is in line with and adds to previous studies, which explored problematic online behaviors being related to video gaming, gambling online, browsing for fun, shopping, and social networking [60,61,62], but not working online. 

The overall percentage among college students and highly educated adults who show problematic internet use was found to be 29.4%, which is also consistent with previous studies in adolescents and adult populations [55,58,63,64]. In other words, if one out of three people is at risk for developing problematic internet use, this calls for preventive actions. Psychological institutes, schools and colleges are recommended to provide coaching and courses to increase individuals’ self-management, in order to avoid problematic internet use. Addressing individuals’ leisure-time internet use can help by raising awareness and skills to regulate internet use, especially in the face of negative feelings and perceived stress.

### 4.2. Perceived Quality of Life in Different Work-Time and Leisure-Time Internet Use Groups

The other main finding of this study was that participants who reported balanced work-time internet use indicated a higher perceived quality of life, compared to the participants who seldom used internet for work and study, with lower than 5 h/w and who spent lots of time on internet use for work with more than 28 h/w. This finding is rather new, as few studies have investigated work-time internet use and perceived quality of life, especially among the population of college students and highly educated adults who frequently use the internet for work and study. This finding supports previous studies that suggest that long work hours are related to a lower quality of life [65,66,67], and that longer work-time internet use may be related to longer work hours [41]. There is no evidence that a lower work-time internet use is related to a lower quality of life in general populations. However, our participants in this study were college students and highly educated adults. Those individuals who seldom use the internet for work may suffer from hidden problems such as a poor work–life balance, and those individuals who use the internet for long periods of time for work may suffer from hidden problems such as too much sitting and too little physical activity, which in turn decreases the quality of life [68,69]. This finding suggests internet use for work should not be too long, and this would prevent the problem of being too sedentary in the context of a working environment.

Specifically, the internet becomes especially important in times of the Coronavirus disease 2019 (COVID-19) crisis, as a considerable amount of work and activities of daily life such as social interactions have been moved online. It helps ensure physical distancing to prevent the virus from spreading, while averting a complete breakdown of all businesses and social connections of individuals and groups. In this unusual period, the time spent on the internet has largely increased. Employers and colleges are recommended to remind their employees and students to avoid potential problematic internet use, while enjoying the advantages of internet. Meanwhile, reducing perceived stress and negative feelings are essential to obtain a higher perceived quality of life. However, this dataset stems from a time prior to the COVID-19 pandemic. While the implications of this study are important for dealing with the COVID-19 pandemic and its aftermath, the main advantage of this study is that the general mechanisms will also be applicable in a time when the COVID-19 pandemic and its aftermath are over. Further studies should research the effects of the COVID-19 pandemic and its aftermath on such mechanisms outlined in this paper; replication and cross-validation are recommended. 

Different leisure-time internet use groups failed to show a significant difference in perceived quality of life in the regression analyses after controlling for socio-demographic variables and work-time internet use. This may be because of although the percentage of problematic internet use was similar to other populations in previous studies [55,58,63,64], the problematic internet users’ score were not very high in this study, which indicated a lower problematic internet use level. The differences between groups with different amounts of leisure-time internet use did, however, demonstrate differences with problematic internet use, which was found to be negatively related with quality of life in this study and in line with previous studies [1,54,55]. Furthermore, perceived quality of life can also be related to other areas of life besides leisure-time internet use, such as sleep, lifestyle, chronic disease, and mental health [41]. The latter can accordingly relate to leisure-time internet use, which should be investigated further in future studies.

### 4.3. Negative Feelings, Perceived Stress, Smoking Status, and Alcohol Assumption Status

Negative feelings and perceived stress were positively related with problematic internet use, which is in line with previous studies [36]. Negative feelings have shown a negative relationship with quality of life, which supports previous findings [5]. The finding that suggested that stress was negatively related to perceived quality of life was in line with previous studies [6,70]; in other words, individuals who feel more stressed also suffer from a lower quality of life. Feeling stressed was no longer found to be a significant predictor of problematic internet use, however, when smoking status and alcohol assumption status were controlled for. This may be due to an interaction of perceived stress and smoking or alcohol status [71]. Previous studies have indicated that smokers may be smoking to relieve perceived stress [72], but this relief is temporary; smoking was found to lead to higher perceived stress overall [72]. Similarly, some individuals exhibiting problematic internet use may spend more time on the internet to escape perceived stress, but end up experiencing higher perceived stress. Future studies should investigate the interactions of feeling stressed and other lifestyle factors for quality of life within a theoretical framework such as the CCAM.

Smoking status failed to show significant relationships with problematic internet use and quality of life, as in previous studies [27], but other studies also found different patterns [7,25]; regular alcohol drinkers showed a higher quality of life compared to nonregular drinkers in this study, which is also in line with and supports previous studies [11,12]. 

### 4.4. Limitations

The results of this study should be interpreted with several limitations in mind. Firstly, the data were mainly collected within Germany, which limits the generalizability of the findings. Secondly, this study relies on self-report measures and a cross-sectional research design, which limits causal conclusions. Moreover, participants in this study were recruited by convenience sampling; future studies are suggested to consider the sample representativeness before data collection. Thirdly, besides work-time and leisure-time internet use, only several variables such as negative feeling were added to the models to predict problematic internet use and quality of life. Other factors such as positive feelings and health status may also impact the results. Further studies should measure and control more confounding variables to exclude their influence, and it may be worth considering further distinguishing social purposes of the internet use to more thoroughly unravel the aspects of leisure-time internet use, and explore how to prevent problematic internet use and its subsequent influences on quality of life. Furthermore, quality of life is related to, but different from life satisfaction, which is measured slightly differently and could also be explored in future studies. Taking these limitations into account, the study still adds to the understanding of mental health in the face of digitalization, and offers suggestions on how to investigate problematic internet use in the future.

## 5. Conclusions

In summary, this study sheds light on using the internet to facilitate daily life and perceived quality of life, while avoiding problematic internet usage. Among college students and highly educated adults, it was found that both work-time and leisure-time internet use can be risk factors for health in terms of problematic internet use and perceived quality of life. Leisure-time internet use rather than work-time internet use was positively associated with problematic internet use. Participants whose work-time internet use was at a balanced level indicated a higher perceived quality of life.

Well-controlled internet use rather than excessive use is recommended, in order to enjoy the convenience brought by the internet and avoid potential drawbacks. Further research can and should build on these findings to ensure a solid knowledge base on which to build an understanding of how internet use can be applied for its advantages, rather than its disadvantages.

## Figures and Tables

**Table 1:** Descriptive statistics of socio-demographics and main research variables.

Variables		*n*	%	Mean (Range)	SD
Gender	Female	266	59.6		
	Male	180	40.4		
Age (yr)		446		25.8 (17–77)	11.6
BMI (kg/m^2^)		446		22.6 (14.3–46.9)	3.8
Married		67	15.0		
Employed and student/in training		434	97.3		
Bachelor and above (%)		232	52.0		
Internet use time (h/w)		446		31.9 (0–116.7)	21.7
Work-time internet use (h/w)		446		17.2 (0–60.0)	14.8
WG1 (<5)		109	24.4	1.9	1.4
WG2 (5–13.99)		99	22.2	8.4	2.5
WG3 (14–27.99)		125	28.0	18.3	3.9
WG4 (28–60)		113	25.3	38.6	9.4
Leisure-time internet use (h/w)		446		14.7 (0–84.0)	13.9
LG1 (<4)		102	22.9	1.8	1.2
LG2 (4–10.99)		122	27.4	7.2	2.0
LG3 (11–20.99)		92	20.6	14.6	2.0
LG4 (21–84)		130	29.1	31.9	13.3
Problematic internet use		446		43.0 (20–80)	11.9
No problematic internet use (score < 50)		315	70.6	36.9 (20–48)	7.4
Problematic internet use (score ≥ 50)		131	29.4	57.8 (50–80)	6.1
Perceived quality of life		446		3.59 (1–5)	0.9
Very poor/Poor		47	10.5		
Neither poor nor good		140	31.4		
Good/Very good		259	58.1		
Negative feelings		446		2.57 (1–5)	0.9
Perceived stress		446		5.24 (2–10)	1.72
Smoking status	Smoker	72	16.1		
	Nonsmoker	374	83.9		
Alcohol assumption status	Regular drinker	179	40.1		
	Nonregular drinker	267	59.9		

WG1: work-time internet use group 1; WG2: work-time internet use group 2; WG3: work-time internet use group 3; WG4: work-time internet use group 4; LG1: leisure-time internet use group 1; LG2: leisure-time internet use group 2; LG3: leisure-time internet use group 3; LG4: leisure-time internet use group 4. BMI = body mass index. SD = Standard deviation.

**Table 2:** Correlation analyses of the main study variables.

Variables	1	2	3	4
1 Work-time internet use				
2 Leisure-time internet use	0.18 **			
3 Problematic internet use	0.06	0.27 **		
4 Perceived quality of life	0.02	−0.02	−0.25 **	

** *p* < 0.001.

**Table 3:** Regression models predicting problematic internet use.

Predictors	Model 1	Model 2	Model 3
	B [95% CI]	B [95% CI]	B [95% CI]
Gender	1.54 [−0.61, 3.69]	1.81 [−0.27, 3.89]	2.30 [0.19, 4.41]
Age	−0.32 [−0.46, −0.19] **	−0.27 [−0.40, −0.14] **	−0.25 [−0.38, −0.12] **
BMI	0.07 [−0.22, 0.36]	0.12 [−0.16, 0.41]	0.09 [−0.19, 0.37]
Marital status	2.89 [−0.97, 6.74]	2.94 [−0.79, 6.68]	2.98 [−0.76, 6.71]
Work status	3.87 [−3.13, 10.87]	4.45 [−2.31, 11.21]	4.53 [−2.19, 11.26]
Work-time Internet use			
WG2 compared WG1	−2.47 [−5.54, 0.61]	−2.91 [−5.89, 0.08]	−2.81 [−5.78, 0.16]
WG3 compared WG1	−2.18 [−5.13, 0.78]	−2.04 [−4.91, 0.84]	−2.02 [−4.90, 0.85]
WG4 compared WG1	−0.38 [−3.37, 2.61]	−1.04 [−3.96, 1.88]	−0.72 [−3.64, 2.20]
Leisure-time Internet use			
LG2 compared LG1	4.13 [1.10, 7.16] *	3.54 [0.61, 6.48] *	3.84 [0.91, 6.78] *
LG3 compared LG1	4.14 [0.85, 7.42] *	4.00 [0.82, 7.17] *	4.08 [0.92, 7.24] *
LG4 compared LG1	6.27 [3.15, 9.38] **	5.87 [2.85, 8.88] **	6.23 [3.20, 9.27] **
Negative feelings		1.27 [−0.11, 2.65]	1.53 [0.14, 2.92] *
Perceived stress		1.30 [0.55, 2.06] **	1.30 [0.54, 2.06] **
Smoking status (nonsmoker = 1)			0.88 [−1.94, 3.70]
Alcohol consumption status (nonregular drinker = 1)			1.28 [−0.88, 3.44]
*R* ^2^	0.16	0.22	0.24
Adjusted *R*^2^	0.14	0.20	0.21

WG1: work-time internet use group 1 (<5 h/w); WG2: work-time internet use group 2 (5–13.99 h/w); WG3: work-time internet use group 3 (14–27.99 h/w); WG4: work-time internet use group 4 (28–60 h/w); LG1: leisure-time internet use group 1 (<4 h/w); LG2: leisure-time internet use group 2 (4–10.99 h/w); LG3: leisure-time internet use group 3 (11–20.99 h/w); LG4: leisure-time internet use group 4 (21–84 h/w). * *p* < 0.05, ** *p* < 0.001. Model 1: socio-demographic variables (gender, age, BMI, marital status, work status). Model 2: model 1 + negative feelings + perceived stress. Model 3: model 2 + smoking status + alcohol consumption status.

**Table 4:** Regression models predicting perceived quality of life.

Predictors	Model 1	Model 2	Model 3
	OR [95% CI]	OR [95% CI]	OR [95% CI]
Work-time Internet use			
WG1 (comparator)	1	1	1
WG2	2.51 [1.41, 4.49] *	2.92 [1.59, 5.35] **	2.78 [1.51, 5.12] **
WG3	1.85 [1.09, 3.14] *	1.75 [1.01, 3.02] *	1.65 [0.95, 2.89]
WG4	1.30 [0.77, 2.21]	1.47 [0.85, 2.56]	1.46 [0.83, 2.56]
Leisure-time Internet use			
LG1 (comparator)	1	1	1
LG2	0.90 [0.52, 1.58]	0.99 [0.56, 1.76]	0.94 [0.53, 1.69]
LG3	0.90 [0.49, 1.66]	0.99 [0.53, 1.86]	0.96 [0.51, 1.81]
LG4	1.04 [0.59, 1.85]	1.15 [0.64, 2.08]	1.09 [0.60, 1.99]
Negative feelings		0.60 [0.46, 0.78] **	0.55 [0.42, 0.73] **
Perceived stress		0.85 [0.73, 0.98] *	0.87 [0.75, 1.01]
Smoking status (nonsmoker = 1)			1.01 [0.58, 1.77]
Alcohol consumption status (nonregular drinker = 1)			0.42 [0.27, 0.65] **

WG1: work-time internet use group 1 (<5 h/w); WG2: work-time internet use group 2 (5–13.99 h/w); WG3: work-time internet use group 3 (14–27.99 h/w); WG4: work-time internet use group 4 (28–60 h/w); LG1: leisure-time internet use group 1 (<4 h/w); LG2: leisure-time internet use group 2 (4–10.99 h/w); LG3: leisure-time internet use group 3 (11–20.99 h/w); LG4: leisure-time internet use group 4 (21–84 h/w). * *p* < 0.05, ** *p* < 0.001. Model 1: socio-demographic variables (gender, age, BMI, marital status, work status). Model 2: model 1 + negative feelings + perceived stress. Model 3: model 2 + smoking status + alcohol consumption status.
